# Improving the Quality of Operation Notes Without a Proforma: An Audit

**DOI:** 10.7759/cureus.116946

**Published:** 2026-09-25

**Authors:** Chibuike Nwachukwu, Charles Ojo, Taiwo Oladimeji

**Affiliations:** 1 General Practice, Aneurin Bevan University Health Board, Newport, GBR; 2 Urology, Airedale NHS Foundation Trust, Keighley, GBR; 3 General Surgery, Royal Free London NHS Foundation Trust, London, GBR

**Keywords:** audit, documentation, note, operation, operative

## Abstract

Aims: An operation note is a medico-legal document and should be accurate and comprehensive. This audit aimed to assess and improve the content of operation notes written in a surgical department at St George’s Hospital, London.

Methods: This was a two-cycle audit involving retrospective review of operation notes in the Breast Surgery Department at St George’s Hospital, London. Sixty-one operation notes from the month of May 2023 were reviewed in the first cycle. Following this, the findings were emailed to clinicians and presented to the department as an intervention to encourage improvement. Verbal reminders were also issued to surgeons in theatre to reinforce the need for detailed operation notes. The audit was repeated after the intervention, reviewing 52 operation notes from the month of December 2023. Data were collated using Excel (StataCorp LLC, College Station, TX, US, USA) and analysed using SPSS (Statistical Package for the Social Sciences; IBM Corp., Armonk, NY, USA). Results are presented in frequency tables.

Results: Post-intervention results demonstrated measurable improvements. Documentation of whether a procedure was emergency or elective increased from 0% in the first cycle to 13.5% in the second cycle. Similarly, recording of anticipated blood loss rose from 0% to 9.6%. Logging of operative diagnosis improved by 19%. There were 12% and 50% improvements in the documentation of details of tissues taken/added/altered and the serial number of implants used, respectively. Although there were modest improvements in documentation of the operating surgeon’s name (1.6%), assistant’s name (5.5%), procedure name (1.6%), and incision details (4.4%), documentation of operative findings and closure technique decreased by 46.7% and 15.5%, respectively.

Conclusion: This audit shows that intervention through presentation and teaching can lead to improvements in the standard of operation notes.

## Introduction

Comprehensive documentation of an operation note is an essential component of good clinical practice. The operation note provides a record of the procedure performed, the operative findings, and the immediate postoperative management plan. It serves as an important source of information for ongoing patient care and facilitates effective communication among healthcare professionals involved in the patient’s management. As a medico-legal document, it must be accurate and comprehensive.

A well-written operative note should contain sufficient detail to provide a clear account of the operation and support continuity of care. Incomplete documentation may result in important clinical information being unavailable to subsequent treating teams and may adversely affect clinical decision-making, patient safety, and the accuracy of the medical record.

The Royal College of Surgeons (RCS) guidance, Good Surgical Practice (2014), sets standards for operation notes to ensure that relevant details are recorded [1]. These include identification of the operating team, name of the procedure performed, operative diagnosis, details of the incision, operative findings, details of tissues taken or altered, perioperative considerations, and postoperative instructions. Despite these guidelines, various international and local audits show that incomplete operation notes remain a persistent problem [2-4].

Studies also demonstrate that highlighting deficiencies in operation note standards through presentations and teaching, as well as the provision of proformas, can improve documentation quality [3-5]. Previous audits have further reported that electronically generated operation notes are of better quality compared with handwritten ones [6-8].

The purpose of this audit was to assess and improve the standard of operation notes in the Breast Surgery Department at St George’s Hospital, London, by identifying deficiencies in compliance with RCS standards and highlighting areas for improvement. This audit involved typed operation notes only, and no proforma was used during the intervention stage, unlike in previous audits [4,9]. It contributes to the existing literature on operation note standards and provides a basis for comparison with subsequent audits involving electronically typed notes and audits where proformas were not used as part of the intervention.

## Materials and methods

This was a two-cycle closed-loop audit involving retrospective review of electronically recorded operation notes in the Breast Surgery Department at St George’s Hospital, London, over a seven-month period. Sixty-one operation notes from May 2023 were reviewed in the first cycle. This number represents the operation notes of all the cases done in the department in the month of May 2023, with no operation notes excluded. Sixteen criteria were extracted from the Good Surgical Practice list of standards for operation notes, and each operation note was assessed against these criteria (Table 1). Parameters such as antibiotic prophylaxis, deep vein thrombosis (DVT) prophylaxis, and implant details were evaluated only when applicable. Several other criteria were excluded because they did not directly apply to this audit. For example, as the operation notes were electronically generated, signature, date, and time were automatically recorded and therefore not included. Criteria relating to complications, additional procedures, and reasons for these were also excluded, as this was a retrospective review.

**Table 1:** Criteria used in audit DVT: deep vein thrombosis

Criteria as per Good Surgical Practice [1]	Criteria used in audit
Date and time	
Elective vs emergency recorded	Elective vs emergency recorded
Name of operating surgeon	Name of operating surgeon
Name of assistant(s)	Name of assistant(s)
Name of theatre anaesthetist	Name of theatre anaesthetist
Name of procedure	Name of procedure
Operative diagnosis	Operative diagnosis
Incision	Incision details
Operative findings	Operative findings
Any problems/complications	
Any extra procedure performed	
Reason for above	
Details of tissues taken/added/altered	Details of tissues taken/added/altered
Identification of any prosthesis/implant used	Identification of any implant used where applicable
Identify serial number and other details of prosthesis/implant used	Identify serial number and other details of implant used where applicable
Details of closure technique	Details of closure technique
Anticipated blood loss	Anticipated blood loss
Antibiotic prophylaxis (where applicable)	Antibiotic prophylaxis (where applicable)
DVT prophylaxis (where applicable)	DVT prophylaxis (where applicable)
Detailed postoperative instructions	Detailed postoperative instructions
Signature	

Completion of the first cycle was followed by an intervention. This intervention involved presenting the findings to consultants, registrars, and core trainees in the department, highlighting the expected standards for operation notes and identifying areas requiring improvement. Surgeons were also reminded during theatre briefings of the need to produce detailed and comprehensive operation notes. The audit was repeated following this intervention, reviewing 52 operation notes from December 2023. This number also represents the operation notes of all the cases performed in the department in the month of December 2023, with no operation notes excluded. Data were collated using Excel (StataCorp LLC, College Station, TX, US, USA) and analysed using SPSS (Statistical Package for the Social Sciences; IBM Corp., Armonk, NY, USA). Results are presented in frequency tables.

## Results

Sixty-one operation notes were reviewed in the first cycle, of which 15 were written by consultants. In the second cycle, 52 operation notes were reviewed, 19 of which were written by consultants. The mean overall compliance increased from 71% in the first audit cycle to 74% in the second; this represents a modest descriptive increase of 3% rather than evidence of a statistically significant improvement, as a formal statistical comparison between audit cycles was not performed. Furthermore, changes in compliance were heterogeneous across individual criteria, with improvement in some domains and deterioration in others.

Documentation of whether the procedure was emergency or elective increased from 0% to 13.5%. Similarly, recording of anticipated blood loss rose from 0% to 9.6%. Recording of operative diagnosis improved by 19%. There were improvements of 12% and 50% in documenting details of tissues taken/added/altered and the serial number of implants used, respectively. Modest improvements were also observed in documentation of the operating surgeon’s name (1.6%), assistant’s name (5.5%), procedure name (1.6%), and incision details (4.4%).

However, there was a decrease in the proportion of operation notes with documentation of operative findings (46.7%), closure technique (15.5%), and DVT prophylaxis (1.9%). None of the operation notes met all the standards. Documentation of antibiotic prophylaxis, where applicable, and postoperative instructions was 100% in both audit cycles. These results are shown in Tables 2, 3.

**Table 2:** Mean compliance

	First cycle	Second cycle	Improvement
Mean compliance (%)	71	74	3

**Table 3:** Compliance with criteria in the two audit cycles DVT: deep vein thrombosis

	First cycle		Second cycle		Change
Criteria	Frequency N = 61	Percentage (%)	Frequency N = 52	Percentage (%)	%
Elective vs emergency recorded	0	0	7	13.5	↑13.5
Name of operating surgeon	60	98.4	52	100	↑1.6
Name of assistant(s)	33	54.1	31	59.6	↑5.5
Name of anaesthetist	43	70.5	34	65.4	↓5.1
Name of procedure	60	98.4	52	100	↑1.6
Operative diagnosis	33	54.1	38	73.1	↑19
Incision details	56	91.8	50	96.2	↑4.4
Operative findings	52	85.2	20	38.5	↓46.7
Details of tissues taken/added/altered	49	80.3	48	92.3	↑12
Identification of any prosthesis/implant used	2 (N = 2)	100	1 (N = 1)	100	0
Identify serial number and other details of prosthesis/implant used	1 (N = 2)	50	1 (N = 1)	100	↑50
Details of closure technique	47	77	32	61.5	↓15.5
Anticipated blood loss	0	0	5	9.6	↑9.6
Antibiotic prophylaxis	16 (N = 16)	100	11 (N = 11)	100	0
DVT prophylaxis	10 (N = 13)	76.9	9 (N = 12)	75	↓1.9
Detailed postoperative instructions	61	100	52	100	0

## Discussion

Audits on operation notes conducted in the UK and internationally have shown that interventions such as presentations, teaching sessions, and the provision of templates or proformas are effective in improving the standard of operation notes [3-5,9]. This audit did not use a proforma as an intervention; instead, the findings from the first cycle were presented to the department, and verbal reminders were issued. Following these interventions, a modest descriptive increase in mean overall compliance was observed, from 71% in the first cycle to 74% in the second cycle. However, as no statistical comparison between cycles was performed and changes across individual criteria were heterogeneous, this increase cannot be attributed solely to the intervention.

This closed-loop audit allowed reassessment of operation note standards following intervention, after awareness of deficiencies identified in the first cycle had been raised within the department. The descriptive mean compliance improved by 3%. A similar study that used comparable interventions without templates or proformas also demonstrated improvement, reporting an 11% increase in mean compliance [10]. These descriptive improvements are modest when compared with findings from other audits reporting mean compliance increases of 28.2% [9] and 22.2% [4], where templates or proformas were used for intervention. The use of structured documentation tools may therefore account for the higher levels of improvement observed in those studies.

Deficiencies in operation note documentation are a common finding across previous audits [3-5,9]. Consistent with these studies, none of the operation notes reviewed in this audit met all RCS standards. An audit of over 1,000 operation notes found that only two operation notes met all RCS criteria [2]. The large sample size and inclusion of multiple UK hospitals in the study may explain this finding, but it also highlights the ongoing need to improve the comprehensiveness of operative documentation.

Documentation of whether a procedure was emergency or elective increased from 0% to 13.5% following intervention. This differs from findings of another audit in which 23.2% of operation notes recorded this information in the first cycle [2]. As this audit was conducted in a breast surgery department where procedures are predominantly elective, surgeons may have overlooked documenting the nature of the procedure. This audit reinforces the importance of recording whether a procedure is elective or emergency.

The importance of documenting anticipated blood loss has been emphasized in previous studies [11]. In the first cycle of this audit, anticipated blood loss was not documented in any operation note. Following intervention, documentation improved to 9.6%. Failure to record anticipated blood loss may adversely affect patient safety, as this information can guide postoperative management.

Post-intervention improvements were also observed in other aspects of documentation, including operative diagnosis (19%), details of tissues taken or altered (12%), and serial numbers of implants used (50%). Identification of the operating surgeon improved from 98.4% to 100%. These findings are consistent with results from both UK and international audits [9,10,12-15].

There was a 4.4% improvement in documentation of incision details. In contrast, a UK audit conducted in 2019 reported a 19% decrease in incision detail documentation, attributing this decline to fatigue associated with handwritten notes [3]. This audit reviewed electronically generated notes, and previous studies have shown that electronic operation notes tend to be more comprehensive than handwritten ones [6-8].

Some areas demonstrated poorer performance in the second cycle, including documentation of operative findings (46.7% decrease), closure technique (15.5% decrease), and DVT prophylaxis (1.9% decrease). These findings contrast with audits reporting improvements in these areas [4,9]. Individual documentation habits and time pressure in theatre may have contributed to these omissions, as surgeons may prioritize recording the procedure itself over other components of the operation note. The smaller sample size in the second cycle may also have influenced these results. Nonetheless, these findings highlight the need for continued efforts to improve operation note standards and ensure documentation is accurate and comprehensive.

Limitations

This audit had several limitations. It was conducted within a single surgical department of a tertiary hospital that comprises multiple surgical specialties, which may limit the generalizability of the findings. The sample size in the second cycle was slightly smaller than that of the first, and both cycles involved relatively small numbers of operation notes. These factors may reduce the robustness of the conclusions. Nevertheless, the overall improvements observed across multiple criteria suggest that the findings remain relevant and indicative of meaningful change.

Recommendations

Given that the development of proformas for operation notes has been shown to improve adherence to documentation standards [4,14], the introduction of a standardized and widely accepted electronic template within the department and potentially across the hospital may further enhance compliance. Electronic recording of operation notes has already demonstrated improved adherence to standards [6-8]. As this audit was conducted in a setting where operation notes are electronically generated, implementation of a standardized electronic template is likely to result in more comprehensive documentation. Further audits following such implementation are recommended, and it would be valuable to compare future findings with those of this study.

We also recommend additional studies exploring the reasons behind poor compliance with the standards set by the RCS. Identifying the underlying causes of inadequate documentation would support the development of sustainable and effective solutions to improve the quality of operation notes.

## Conclusions

Several publications have proposed different approaches to improving the standard of operation notes. This audit demonstrates that departmental presentation of identified deficiencies, combined with verbal reminders to surgeons during theatre briefings, can lead to some improvements in documentation quality. However, the use of multiple intervention strategies including the incorporation of proformas appears more effective and is likely to result in greater improvement of operation note standards.
